# Live‐cell CRISPR imaging in plants reveals dynamic telomere movements

**DOI:** 10.1111/tpj.13601

**Published:** 2017-07-14

**Authors:** Steven Dreissig, Simon Schiml, Patrick Schindele, Oda Weiss, Twan Rutten, Veit Schubert, Evgeny Gladilin, Michael F. Mette, Holger Puchta, Andreas Houben

**Affiliations:** ^1^ Leibniz Institute of Plant Genetics and Crop Plant Research (IPK) Gatersleben 06466 Seeland Germany; ^2^ Botanical Institute Karlsruhe Institute of Technology POB 6980 76049 Karlsruhe Germany; ^3^ Present address: King Abdullah University of Science & Technology Thuwal 23955‐6900 Saudi Arabia

**Keywords:** CRISPR–dCas9, live cell imaging, telomeres, chromatin dynamics, nucleus, *Nicotiana benthamiana*, technical advance

## Abstract

Elucidating the spatiotemporal organization of the genome inside the nucleus is imperative to our understanding of the regulation of genes and non‐coding sequences during development and environmental changes. Emerging techniques of chromatin imaging promise to bridge the long‐standing gap between sequencing studies, which reveal genomic information, and imaging studies that provide spatial and temporal information of defined genomic regions. Here, we demonstrate such an imaging technique based on two orthologues of the bacterial clustered regularly interspaced short palindromic repeats (CRISPR)–CRISPR associated protein 9 (Cas9). By fusing eGFP/mRuby2 to catalytically inactive versions of *Streptococcus pyogenes* and *Staphylococcus aureus* Cas9, we show robust visualization of telomere repeats in live leaf cells of *Nicotiana benthamiana*. By tracking the dynamics of telomeres visualized by CRISPR–dCas9, we reveal dynamic telomere movements of up to 2 μm over 30 min during interphase. Furthermore, we show that CRISPR–dCas9 can be combined with fluorescence‐labelled proteins to visualize DNA–protein interactions *in vivo*. By simultaneously using two dCas9 orthologues, we pave the way for the imaging of multiple genomic loci in live plants cells. CRISPR imaging bears the potential to significantly improve our understanding of the dynamics of chromosomes in live plant cells.

## Significance Statement

Visualizing the spatio‐temporal organisation of the genome can improve our understanding of how chromatin structure and function are intertwined. We developed a toolkit to visualize defined genomic sequences in living plant cells based on fluorescence‐tagged CRISPR‐dCas9 and demonstrated its ability to reveal dynamic telomere movements.

## Introduction

The spatial and temporal organization of genomes is important for maintaining and regulating cell functions such as gene expression, DNA replication and repair, and the proper segregation of genetic material during cell division. Elucidating how the genome is spatiotemporally organised inside the nucleus is imperative to our understanding of how genes and non‐coding sequences are regulated during development. Mapping the functional organization of the genome can be achieved by visualizing interactions between different genomic elements in living cells. Although fluorescence‐tagged nuclear proteins can be readily imaged in living plant cells, the *in vivo* visualization of defined DNA sequences is technically difficult. Fluorescent *in situ* hybridization (FISH), a well‐established tool to map DNA sequences, relies on fixed tissue samples and cannot be used to visualize dynamic processes. Furthermore, FISH requires cell fixation and a DNA denaturation step that may result in an altered chromatin structure (Kozubek *et al*., 2000; Boettiger *et al*., 2016).

Live cell labelling of specific genomic loci has been achieved by the application of a directly repeated *lac* operator sequence and its detection with a GFP‐lacI repressor protein (Kato and Lam, 2001). However, this system is based on the random insertion of an artificial sequence into the genome. Live imaging of endogenous genomic regions became possible with the application of fluorescence‐tagged zinc‐finger proteins. A zinc‐finger GFP protein was designed to recognize a 9‐bp sequence within the centromeric 180‐bp tandem repeat of *Arabidopsis thaliana* (Lindhout *et al*., 2007). Despite the numerous uses of engineered zinc‐finger proteins for genome editing, the potential of this technology has not yet been fully exploited for chromatin imaging. The discovery of the *Xanthomonas*‐based DNA binding domain (Boch *et al*., 2009), which can be engineered to bind specific DNA sequences, initiated the development of transcription activator‐like effectors (TALEs) fused with fluorescence proteins (Ma *et al*., 2013). Although fluorescently labelled TALEs were successfully used to trace genomic loci in non‐plant organisms (reviewed in Chen *et al*., 2016a), their application in plants has only recently been shown by Fujimoto *et al*., (2016).

The discovery of the type‐II clustered regularly interspaced short palindromic repeats (CRISPR) system derived from *Streptococcus pyogenes*, has revolutionized the field of targeted genome editing in eukaryotes (Jinek *et al*., 2012). Cas9 nuclease‐based genome engineering has become a routine technology for many plant species (reviewed in Pacher and Puchta, 2017); however, the full potential of this technology reaches far beyond the controlled induction of mutations. The Cas9 nuclease can be transformed by two point mutations into a site‐specific DNA‐binding protein, which can be fused with different protein domains. Thus, it should in principle be possible to target any kind of enzymatic activity to any genomic site of interest (Puchta, 2016a). Recently, nuclease‐deficient derivatives (dCas9) were used to modify gene expression in many model organisms, including plants (Qi *et al*., 2013; Piatek *et al*., 2015). Furthermore, by fusing dCas9 with GFP, the CRISPR–dCas9 system has been used to label genomic loci in live mammalian cells (Chen *et al*., 2013; Anton *et al*., 2014). Multicolour CRISPR–dCas9 imaging became possible with the application of dCas9 orthologues from different bacterial species, like *Neisseria meningitidis* (NmCas9), *Streptococcus thermophilus* (St1Cas9), and *Staphylococcus aureus* (SaCas9) (Ma *et al*., 2015; Chen *et al*., 2016b). The discovery of the Cas9‐like activities of the Cpf1 proteins derived from *Acidaminococcus* and *Lachnospiraceae* (Zetsche *et al*., 2015) may further expand the palette of multicolour CRISPR–dCas9 imaging. More importantly, orthologues of *S. pyogenes* (Sp‐Cas9), such as St1‐Cas9 and Sa‐Cas9, have been confirmed to be functional in plants (Steinert *et al*., 2015).

In the current study, we describe the development of CRISPR–dCas9 for live cell imaging in plants based on two Cas9 orthologues derived from *S. pyogenes* (Sp‐dCas9) and *S. aureus* (Sa‐dCas9). We demonstrate reliable imaging of telomere repeats in living cells of *Nicotiana benthamiana* and pave the way for the potential visualization of multiple genomic loci. Furthermore, we show that CRISPR–dCas9 can be combined with fluorescence‐labelled proteins to investigate DNA–protein interactions *in vivo*.

## Results & Discussion

### CRISPR–dCas9 enables the visualization of tandem repeats in live plant cells

**Figure 1:**
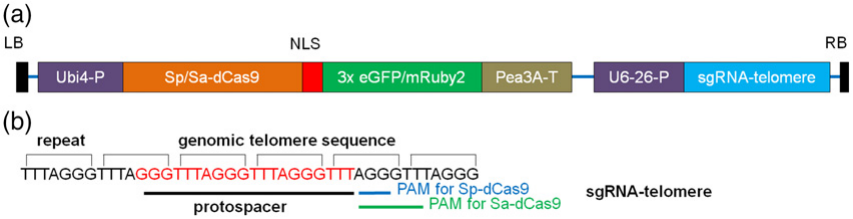
Structure of the CRISPR–dCas9 construct. (a) Transcription of Sp/Sa‐dCas9 was initiated by the parsley ubiquitin 4 promoter and terminated by the pea 3A terminator. An SV40 NLS DNA sequence was used for nuclear localization of dCas9. Transcription of the sgRNA scaffold was initiated by the *Arabidopsis* ubiquitin 6 promoter. (b) Protospacer design for Sp‐dCas9 and Sa‐dCas9 to target telomere DNA sequence. Target sequence is shown in red. The NGG protospacer adjacent motif (PAM) for Sp‐dCas9 is indicated in blue, whereas *NNGRRT* PAM for Sa‐dCas9 is indicated in green.

To establish live cell imaging by CRISPR–dCas9 in plants, we introduced a point mutation in the RuvC1 and HNH nuclease domains (D10A and H841A) in two Cas9 orthologues derived from *S. pyogenes* (Sp‐Cas9) and *S. aureus* (Sa‐Cas9), which were previously used for targeted mutagenesis in *A. thaliana* (Fauser *et al*., 2014; Steinert *et al*., 2015), rendering the Cas9 protein catalytically inactive. Multiple copies of fluorescence proteins, either eGFP or mRuby2, were fused to the C‐terminal end of each dCas9 variant (Figure 1a).

To test the functionality of CRISPR–dCas9 for live cell imaging in plants, we imaged the telomeres of *Nicotiana benthamiana* in leaf cells. *Nicotiana* telomeres are composed of arrays of TTTAGGG repeats that are 60–160 kb in length (Fajkus *et al*., 1995). These tandemly repeated DNA sequences allow the binding of many dCas9 proteins at the same locus by a single single‐guide (sg) RNA sequence. To target telomeric repeats we constructed a 20‐nucleotide sgRNA that was complementary to the TTTAGGG telomere sequence, starting with a ‘G’ at the 5′ end (sgRNA‐telomere; Figure 1b). The 5′‐G nucleotide was selected as the *A. thaliana* U6‐26 promoter employed requires it to initiate transcription (Belhaj *et al*., 2013).

**Figure 2:**
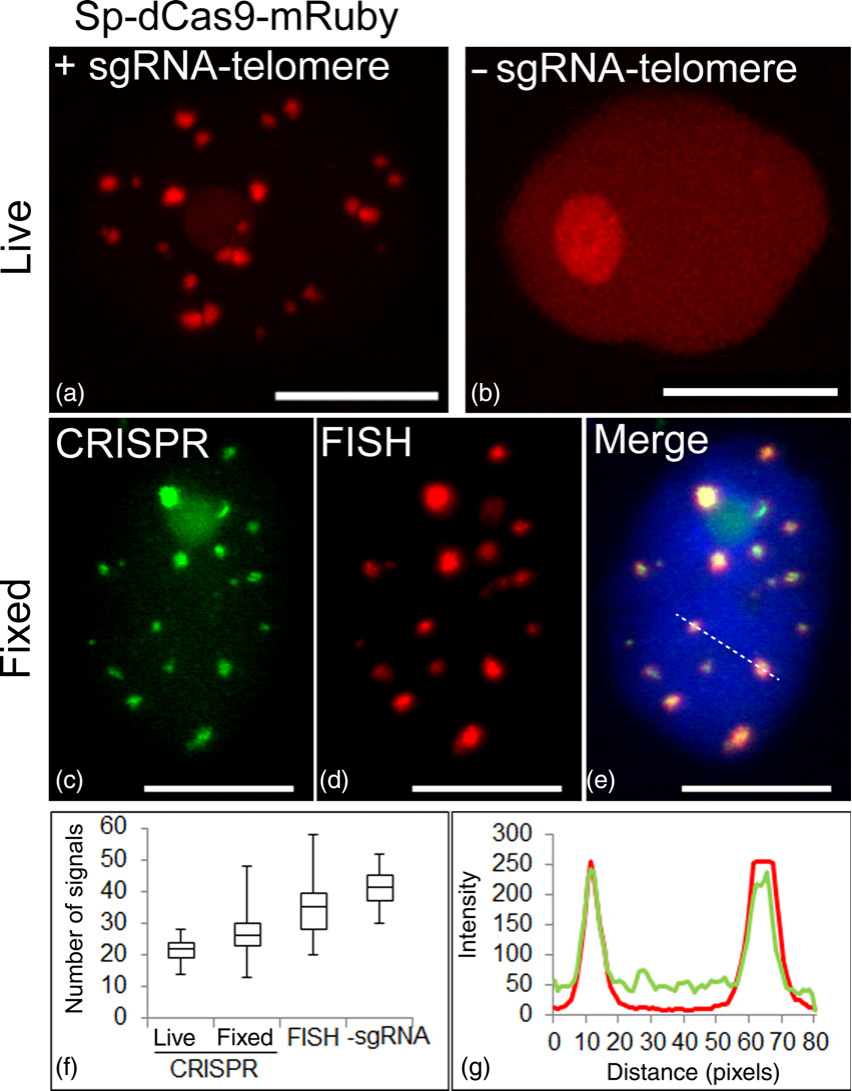
Live imaging of telomeres by CRISPR–dCas9. (a) Sp‐dCas9‐mRuby and sgRNA ‐telomere were used for live imaging of telomeres in *N. benthamiana* leaf cells during interphase (*n* = 50). (b) As a negative control, the telomere sgRNA was omitted. (c–e) Immunofluorescence staining against Sp‐dCas9‐eGFP (c), combined with FISH against telomeres (d), and overlain to confirm co‐localization. Nucleus is counterstained with DAPI (in blue) (e). (f) Whisker box plot showing the efficiency of Sp‐dCas9 for telomere labelling (*n* = 50 nuclei). CRISPR live refers to the number of signals in live leaf nuclei, whereas CRISPR fixed refers to the number of signals in isolated nuclei after fixation. –sgRNA indicates the number of telomeres counted after transformation of dCas9 without the sgRNA‐telomere. (g) Intensity plot showing a positive correlation between FISH (red) and CRISPR imaging (green) regarding the size and intensity of hybridization signals (indicated by the dotted line in panel e) (*n* = 30). Scale bars: 10 μm.

We used infiltration by *Agrobacterium tumefaciens* to transiently express Sp‐dCas9 and sgRNA‐telomere in leaf cells. As a negative control, the same dCas9 construct was infiltrated without a specific sgRNA. After 2–4 days, bright fluorescence puncta were observed in addition to a weak nonspecific background labelling of the nucleus and in particular of the nucleolus (Figure 2a). A similar nonspecific labelling of the nucleolus caused by CRISPR–dCas9 was observed in previous studies (Chen *et al*., 2013; Zhou *et al*., 2017). In negative controls, only a weak nonspecific labelling of the nucleus was observed (Figure 2b). In live interphase nuclei, we detected an average of 21.75 telomere signals by CRISPR imaging (*n* = 50 nuclei). To confirm the telomere specificity of the fluorescence signals and to quantify the efficiency of dCas9 telomere labelling, we analysed the co‐localization of dCas9 and telomeres by immunofluorescence and fluorescent *in situ* hybridization (FISH; Figure 2c–e). On average, 27 signals were detected by immunofluorescence labelling of dCas9, which amounts to 78% of all telomeres detected by FISH (Figure 2e, f). We observed an average of 35 telomere FISH signals, which indicates a certain degree of telomere clustering as the expected number of telomeres based on a chromosome complement of 2*n* = 38 would be 76 in 2C nuclei (Appendix S1). Notably, a similar localization pattern was observed in wild‐type leaf interphase nuclei (Appendix S2), although we detected a higher number of telomere signals (average = 53, *n* = 30). We then used dCas9 without introducing a specific sgRNA as a control, and detected an average of 42 signals (*n* = 30). As the detectable number of telomeres appears to be highly variable in *N. benthamiana* leaf nuclei, we conclude that mainly nuclei with clustered telomeres were visualized in our experimental system. A higher number of CRISPR–dCas9 signals were observed in fixed cells after immunofluorescence labelling compared to live cells, which might be a result of the improved detection efficiency of the GFP antibody. The intensity of individual hybridization signals most likely varied because of chromosome‐specific differences in telomere repeat number and fusion of chromosome ends. Importantly, we observed a positive correlation (ρ = 0.84, *r* ^²^ = 0.7, *n* = 30) between FISH and CRISPR imaging regarding the intensity and size of hybridization signals (Figure 2g).

### Imaging of telomeres by CRISPR–dCas9 reveals long‐range telomere dynamics

We then attempted to explore the possibility of visualizing telomere movement in a live nucleus by CRISPR imaging. To visualize the nuclear envelope in addition to telomeres, we used the nuclear egress protein of the human cytomegalovirus pUL50 fused to GFP, which was previously shown to localize to the *N. benthamiana* nuclear envelope (Lamm *et al*., 2016, Appendix S3). Individual nuclei were observed *in vivo* for a total of 30 min, and *z*‐stacks were acquired in 1‐min intervals. We observed stable fluorescence over the entire period of time.

**Figure 3:**
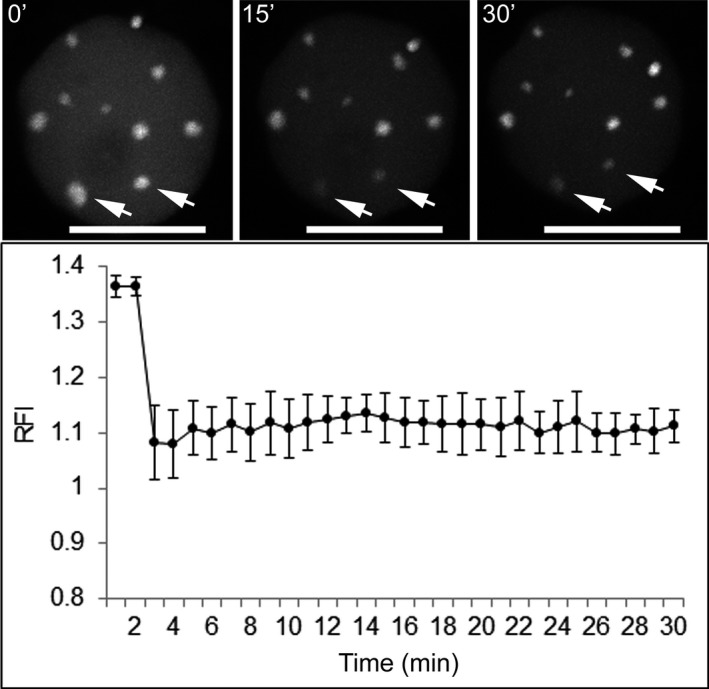
Fluorescence recovery after photobleaching (FRAP) analysis demonstrates a stable association of dCas9 with the target sequence during interphase. FRAP experiments were conducted on three individual nuclei. A region of interest was bleached (indicated by arrows) and the fluorescence intensity was compared with the background fluorescence intensity to determine the relative fluorescence intensity (RFI). Error bars represent standard deviations, based on three biological replicates. Scale bars: 10 μm.

To investigate whether there is a stable association of dCas9 with its target sequence or rapid turnover, we conducted fluorescence recovery after photobleaching (FRAP) experiments. After bleaching individual dCas9 clusters of three nuclei, we found no significant fluorescence recovery over a period of 30 min (Figure 3). This indicates a stable association of dCas9 with its target sequence, which is in agreement with previous reports showing an average target residence time of more than 3 h in mammalian cells (Ma *et al*., 2016; Qin *et al*., 2017).

**Figure 4:**
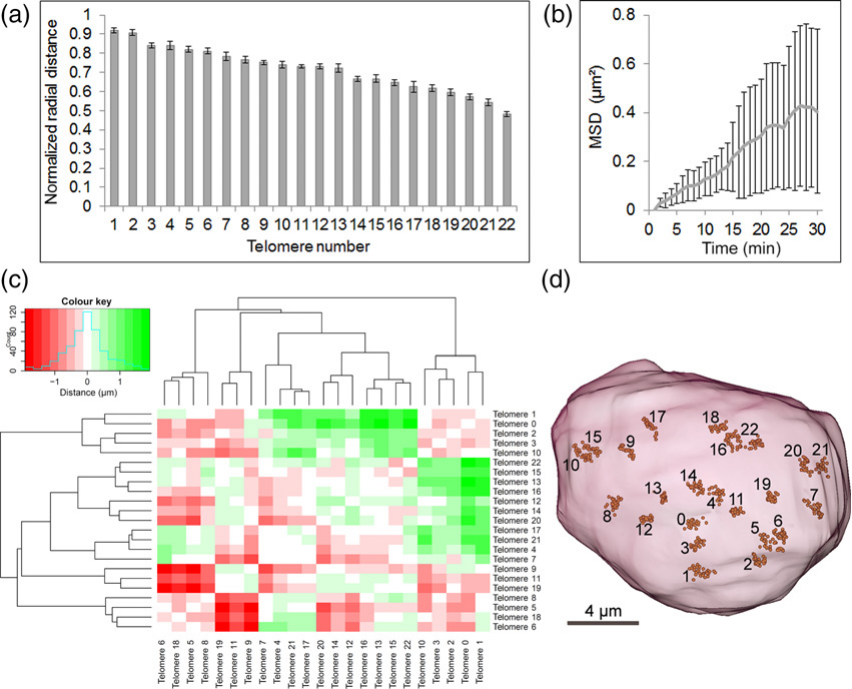
CRISPR–dCas9 enables the 3D tracking of telomeres and reveals long‐range movements in interphase nuclei. (a) Normalized radial distance (NDR) of telomeres of a representative nucleus. An NDR of 0 indicates localization in the centre of the nucleus, whereas an NDR of 1 indicates localization at the nuclear envelope. Error bars represent standard deviations, based on measurements conducted at different time points (1–30 min). Telomere number represents individual telomere signals in a live nucleus. (b) Mean square displacement (MSD) in μm² was measured in 12 live nuclei with a total of 181 telomere signals over a period of 30 min. Error bars represent standard deviations. (c) Heat map showing changes in intertelomere distance over a period of 30 min in a representative nucleus. Colours represent increased (green) and decreased (red) intertelomere distances, by up to 2 μm. (d) Simultaneous visualization of 3D telomere locations in the same nucleus as in (c) from all time points after rigid registration to a reference system of coordinates given by the first time point.

Several dynamic subcellular movements were observed. First, entire nuclei showed movements in all three dimensions over time. Within those nuclei, however, telomeres tended to follow these movements, but also changed their position relative to each other (Appendix S4) and were localized in proximity to the nuclear envelope, which was shown by a mean normalized radial distance of telomeres of 0.74 (Figure 4a; Appendix S5). To quantify these dynamics, we tracked the spatial movements of telomeres over the entire period of time and measured their mean square displacement (MSD; Figure 4b; Appendices S6 and S7). By tracking single telomere clusters, we observed confined diffusion of telomere clusters as well as long‐range movements, which results in a high standard deviation of the MSD. To reveal these variations in a representative nucleus, we calculated absolute changes in intertelomere distance over time (Figure 4c, d). Within 30 min, individual telomeres changed their distance from each other by up to ±2 μm (average diameter of the nucleus = 15.12 μm, *n* = 12). Compared with an average intertelomere distance of 8.1 μm, these changes can amount to a maximum of 24.7%. Similar observations were made previously in *A. thaliana* by labelling telomeres via fluorescent TALEs (Fujimoto *et al*., 2016). In contrast to Fujimoto *et al*. (2016), however, we did not observe an active formation of telomere clusters. This might be related to differences in interphase telomere dynamics between *A. thaliana* root cells and *N. benthamiana* leaf cells rather than CRISPR–dCas9 having a negative effect on telomere dynamics, as both MSD curves are similar. Telomere dynamics during interphase might be related to the transcription of telomeric tandem repeats (Koo *et al*., 2016), telomerase activity (Schrumpfova *et al*., 2016), or positional silencing by telomeres (Gottschling *et al*., 1990; Nimmo *et al*., 1994; Cryderman *et al*., 1999). Similar long‐range chromatin dynamics of specific interstitial chromosomal regions were previously described based on fixed *A. thaliana* cells (Schubert *et al*., 2014). Our results reveal that long‐range chromatin movements can occur over a short period of time, which we suggest is highly relevant for chromatin conformation capture studies (reviewed in Bonev and Cavalli, 2016) aiming to look at such interactions. We conclude that CRISPR–dCas9 is a robust system to reveal the dynamics of telomeres in live plant cells.

### Visualization of DNA–protein dynamics at plant telomeres

As CRISPR–dCas9 is a tool to visualize specific DNA sequences, it can be combined with other methods, e.g. fluorescence‐labelled proteins, to study the dynamics of DNA–protein interactions. As an example, we attempted to visualize telomeric DNA by CRISPR–dCas9 and the telomeric repeat binding protein 1 (TRB1) in live leaf cells of *N. benthamiana*. TRB1 was previously found to be located at plant telomeres and to interact with telomerase reverse transcriptase (TERT), although not all telomeres are bound by TRB1 (Dvorackova *et al*., 2010; Schrumpfova *et al*., 2014). Interestingly, telomeres in yeast, ciliates, mammals and plants may form 3′ overhangs; however, in plants, blunt‐ended telomeres and telomeres with 3′ overhangs may appear in the same cell, and only telomeres exhibiting 3′ overhangs are bound by TRB1.

**Figure 5:**
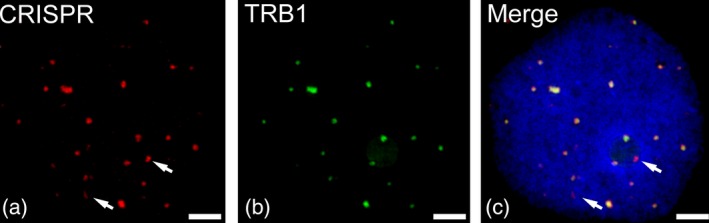
Simultaneous visualization of telomeric DNA by CRISPR–dCas9 and the GFP‐tagged telomeric repeat binding protein 1 (TRB1). (a) Immunofluorescence staining against Sp‐dCas9‐mRuby2. (b) Immunofluorescence staining against TRB1‐GFP. (c) Overlay showing almost complete co‐localization, except for putative blunt‐ended telomeres (indicated by arrows, nucleus is counterstained with DAPI (in blue). Scale bars: 2 μm.

We simultaneously expressed Sp‐dCas9‐mRuby, sgRNA‐telomere and TRB1‐GFP in leaf cells to visualize the dynamic relationship between the telomeric repeats of all chromosomes, and the proportion that are bound by TRB1. On average, we detected 30 CRISPR–dCas9 signals resembling telomeres, 26.3 (87.6%) of which were simultaneously bound by TRB1 (Figure 5). This indicates that most telomeres in *N*. *benthamiana* form 3′ overhangs and only a small proportion of blunt‐ended telomeres are present during the interphase. Our results demonstrate that CRISPR–dCas9 can be used to visualize specific DNA sequences in combination with fluorescently tagged proteins interacting with those DNA sequences. We hypothesize that this principle can be expanded to investigate spatiotemporal gene expression patterns, e.g. by visualizing DNA sequences and transcription factors, as well as other DNA–protein interactions, such as the loading of specific histone variants to certain genomic regions (e.g. CENH3 and centromeric DNA).

### Comparing the efficiency of two dCas9 orthologues for the imaging of telomeres

**Figure 6:**
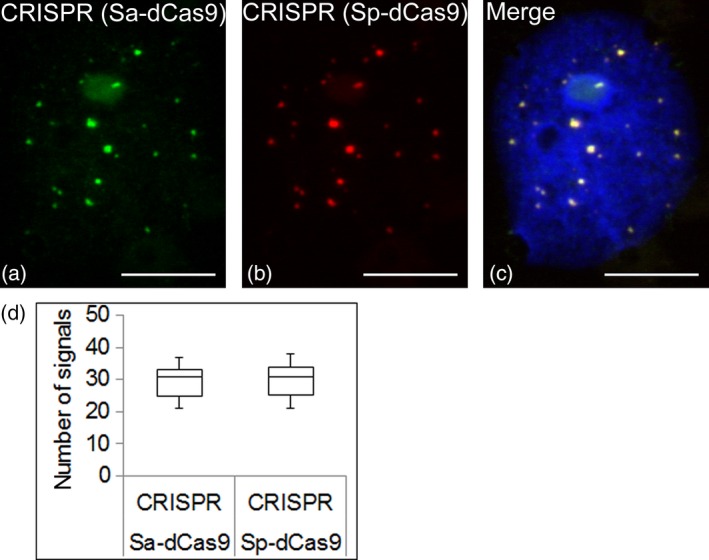
Comparison of Sa‐dCas9 and Sp‐dCas9. Telomeres were visualized by the simultaneous application of two dCas9 orthologues (Sa‐dCas9 and Sp‐dCas9). (a) Immunofluorescence staining against Sa‐dCas9‐eGFP. (b) Immunofluorescence staining against Sp‐dCas9‐mRuby2. (c) Overlay showing complete co‐localization. Nucleus is counterstained with DAPI (in blue). (d) Quantification of the number of telomere signals observed by two different dCas9 orthologues (*n* = 18). Scale bars: 10 μm.

In addition to Sp‐dCas9 derived from *Streptococcus pyogenes*, we used Sa‐dCas9 derived from *Staphylococcus aureus* to visualize telomeres, compare their efficiency and potentially pave the way for the simultaneous imaging of multiple genomic loci in plants. The protospacer‐adjacent motif (PAM) required by Sa‐dCas9 (NNGRRT) allowed us to use the same sgRNA as for Sp‐dCas9. In contrast to Sp‐dCas9, the sgRNA scaffold sequence of Sa‐dCas9 as well as the size of the complex itself differs (Sa‐dCas9 = 1064 amino acids versus Sp‐dCas9 = 1380 amino acids), which could have an effect on its efficiency (Chen *et al*., 2016b). In previous experiments, we showed that in plant cells Sp‐Cas9 and Sa‐Cas9 only form a complex with their respective sgRNAs, and not with the sgRNA of the other orthologue (Steinert *et al*., 2015). Compared with Sp‐dCas9 detecting 78% of all telomeres, using Sa‐dCas9 we were able to detect 85.5% of all telomeres when both variants were analysed separately. We then used both variants Sp‐dCas9‐mRuby2 and Sa‐dCas9‐eGFP simultaneously to visualize telomeres and demonstrate the potential application of different dCas9 orthologues for multiple genomic loci. When combined, both variants showed almost complete co‐localization, indicating no significant difference in their efficiency to detect telomeres (Figure 6). We conclude that both Sp‐dCas9 and Sa‐dCas9 can be used simultaneously to visualize tandem repeats in live plant cells. Thus, by using different Cas9 or Cpf1 orthologues fused to different fluorescence proteins, multidimensional live imaging in single cells might become a reality in the long run (Puchta, 2016b). For tandem repetitive sequences, a single sgRNA may be sufficient for CRISPR imaging, whereas labelling of non‐repetitive loci (spanning a 5‐kb region) may require the simultaneous expression of at least 30 sgRNAs (Chen *et al*., 2013). More recently, an entire human chromosome was visualized with CRISPR imaging by using at least 485 non‐repetitive sgRNAs at the same time (Zhou *et al*., 2017). An alternative approach is to tether fluorescent RNA binding proteins to the sgRNA through aptamer fusions, thereby transforming the sgRNA into a scaffold RNA that contains information about the target locus and the type of fluorescence (Shao *et al*., 2016). These recent developments may further improve CRISPR imaging in plants and potentially enable us to visualize single genomic loci.

## Experimental procedures

### T‐DNA construction

All constructs are based on our previously described vector pCAS9‐TPC (Fauser *et al*., 2014). For the two Cas9 orthologues from *Streptococcus pyogenes* (Sp) and *Staphylococcus aureus* (Sa), respective dCas9 versions were generated by two consecutive rounds of site‐directed mutagenesis, thereby introducing the two point mutations (D10A/H840A for Sp‐dCas9 and D10A/N580A for Sa‐dCas9). Plasmids encoding for eGFP (pSiM24‐eGFP) and mRuby2 (pcDNA3‐mRuby2) were obtained from Addgene (http://www.addgene.com). dCas9 and fluorescence protein (FP) sequences were generated with primers containing homologous flanks for subsequent Gibson Assembly cloning into the pCAS9‐TP backbone (for a full list of primers, see Appendix S8). The stop codons of the dCas9 and the first two FP sequences were removed to generate a continuous open reading frame (ORF), harbouring the respective dCas9 orthologue followed by a threefold fusion of the appropriate FP sequence. The dCas9 sequence and the FP fusion as well as the single FP sequences were linked via a GS‐rich linker, respectively. Protospacers were allowed to self‐anneal and the resulting 4‐bp overhangs were used for subsequent ligation into the respective pChimera vector via *Bbs*I restriction sites. The customised RNA chimeras were then ligated into the respective dCas9 vectors via *Mlu*I restriction sites. The Cas9 constructs developed in this study are available on request to HP.

### Protospacer design

Protospacer sequences were selected based on the respective PAM sequence of each dCas9 orthologue, namely SpdCas9 and SadCas9, and synthesized as oligonucleotides with appropriate 4‐bp 5′ overhangs for cloning into the respective pChimera vector. The telomere‐specific protospacer (5′‐GGGTTTAGGGTTTAGGGTTT‐3′) is based on the Arabidopsis‐type telomere repeat sequence 5′‐(TTTAGGG)(n)‐3′. As a result of the presence of both Sp‐dCas9 (5′‐NGG‐3′) and Sa‐dCas9 (5′‐NNGRRT‐3′) PAM sequences in the telomere repeat sequence, both variants were used to label telomeres, which allowed us to compare these two orthologues.

### Transient transformation of *N. benthamiana*

All CRISPR–dCas9 constructs, TRB1‐GFP (Schrumpfova *et al*., 2014) and pUL50‐GFP (Lamm *et al*., 2016) were separately transformed into *Agrobacterium tumefaciens* strain GV3101 by electroporation. Agrobacteria containing Sp‐dCas9, Sa‐dCas9 and pUL50‐GFP were cultured in YEB medium (5 g l^‐1^ beef extract, 1 g l^‐1^ yeast extract, 5 g l^‐1^ peptone, 5 g l^‐1^ sucrose, 300 mg l^‐1^ MgSO4, 20 g l^‐1^ agar) containing spectinomycin (100 μg ml^−1^) and rifampicin (50 μg ml^−1^). For TRB1‐GFP, Agrobacteria were cultured in YEB medium containing kanamycin (100 μg ml^−1^) and rifampicin (50 μg ml^−1^). The transient transformation of *N. benthamiana* leaf cells was performed as described in Phan and Conrad (2016). For the transformation of multiple constructs, bacterial cultures with an OD_600_ between 1.0 and 1.3 were mixed in a 1:1 ratio prior to transformation. Plants were analysed 2–4 days after infiltration.

### Immunofluorescence analysis and fluorescence *in situ* hybridization

Two or three days after leaf infiltration, nuclei were extracted by chopping a 1‐cm^²^ piece of leaf tissue in 1 ml of chromosome isolation buffer (Dolezel *et al*., 2007) using a razor blade followed by filtration through a 35‐μm nylon mesh and subsequent centrifugation onto a microscopic slide at 400 rpm for 5 min (Shandon CytoSpin3, https://gmi-inc.com). To confirm the specificity of each dCas9 construct, we conducted immunofluorescence staining against eGFP and mRuby2 in combination with fluorescence *in situ* hybridization (immuno‐FISH) against telomeres. Immuno‐FISH was performed as described by Ishii *et al*. (2015). eGFP was detected with a polyclonal GFP antibody (GFP antibody Dylight 488; Rockland, https://www.rockland-inc.com) in a 1:2500 dilution. mRuby2 was detected with a primary RFP antibody [RFP antibody (5F8), 1:1000 dilution; Chromotek, http://www.chromotek.com] generated in rats followed by an anti‐rat secondary antibody (ab96889; abcam, http://www.abcam.com). To detect telomeres via FISH, we used 5′‐Cy5 labelled oligonucleotides composed of the same DNA sequence as the respective protospacer (sgRNA‐telomere, 5′‐GGGTTTAGGGTTTAGGGTTT‐3′). A final probe concentration of 0.33 μm was used. The correct telomeric localization of our FISH probe was validated by testing on *N. benthamiana* chromosomes (Appendix S1) prepared from flower buds using a protocol described by Sanchez Moran *et al*. (2001).

### Microscopic analyses

To analyse co‐localization between CRISPR–dCas9 imaging and FISH signals, images were acquired with an epifluorescence microscope (BX61; Olympus, https://www.olympus.com) using a cooled charge coupled device (CCD) camera (Orca ER; Hamamatsu, http://www.hamamatsu.com), and analysed with imagej. A total of 50 nuclei were analysed by immuno‐FISH to determine the efficiency of dCas9 to detect telomeres. The number of *in vivo* dCas9 signals was counted in 50 live nuclei to determine the *in vivo* labelling efficiency. To analyse the co‐localization of telomeres visualized by Sp‐dCas9 and TRB1, a total of 43 nuclei were analysed by epifluorescence microscopy. Structured illumination microscopy (SIM) was applied to a representative sample using a 63×/1.4Oil Plan‐Apochromat objective of an Elyra PS.1 microscope system with zen software (Carl Zeiss, https://www.zeiss.com). Image stacks were captured separately for each fluorochrome using appropriate excitation and emission filters. Maximum intensity projections were generated from the stacks of SIM sections through the specimens in zen (3D rendering based on SIM image stacks was carried out using imaris 8.0; Bitplane, http://www.bitplane.com).

For live cell imaging of telomeres, fluorescence signals were analysed 2–4 days after infiltration with *A. tumefaciens* (see transient transformation of *N. benthamiana*) by a LSM780 (Carl Zeiss). Infiltrated leaf areas were cut and mounted onto a microscopic slide. The distribution of fluorescence signals within the nucleus was recorded as *z*‐stacks (*n* = 50 nuclei). For a co‐distribution analysis, probes were excited with dual 488‐ and 561‐nm laser lines in combination with a 488/561‐nm beam splitter. eGFP emission was detected over a range of 490–540 nm, and mRuby2 emission was detected over a range of 570–620 nm. Photospectrometric analysis of the fluorescence signal by means of the META detector confirmed the identity of GFP and mRuby. The turnover of Sp‐dCas9‐eGFP telomeric signals was investigated by FRAP analysis. After two pre‐scans a region of interest of variable size was bleached. To achieve appropriate bleaching, the 488‐nm laser line was set at 100% power with 25 iterations at scan speed 7. Fluorescence intensity was followed over 30 min in 1‐min intervals.

### Tracking of telomere signals and 3D image analysis

Telomere tracking based on time‐lapse *z*‐stacks was conducted with imaris 8.0 (Bitplane). Brightness was manually adjusted to detect all telomere clusters. Tracking was performed using the autoregression motion algorithm, with a maximum distance of 20 μm and a maximum gap size of 3. Afterwards, the coordinates (*x*,* y*,* z*) of each spot at all time points were used to quantify telomere movements. Intertelomere distances were calculated for all telomeres of a representative nucleus based on differences in distance between time point 1 and time point 30. For this purpose, an intertelomere distance matrix was generated for time point 1 (matrix1) and time point 30 (matrix30). We then calculated the change in intertelomere distance by subtracting matrix1 from matrix30. The resulting matrix was then visualized as a heat map generated in rstudio using the heatmap2 function of the gplots package. Distances are presented in μm and visualized by two different colours, indicating an increase (green) or decrease (red) of intertelomere distance over time.

The 3D stacks of 12 live nuclei were semi‐automatically segmented and triangulated surfaces of nuclear boundaries were generated in amira 4.1 (Mercury Computer Systems, https://www.mrcy.com). To account for relative nuclear movements (i.e. translations, rotations), 3D point clouds of telomere mass centres from subsequent time points (*t* > 0) were rigidly registered to the reference system of coordinates given by the first time point (*t* = 0) using absolute orientation quaternions (Horn, 1987). To characterize the intranuclear telomere motion, the MSD of telomeres relative to their initial position (*t* = 0) was calculated as

(1) $$\text{MSD}\left(t\right)=\frac{1}{N}\sum_{i=1}^{N}{\left(R_{i}\left(t\right)-R_{i}\left(0\right)\right)}^{2}$$

where *R*_*i*_(*t*) denotes the radius vector of the *i*‐th registered telomere in the reference system of coordinates at time point *t* > 0. The intranuclear position of telomeres was quantified in three representative nuclei by the normalized radial distance (NRD):

(2) NRD=TN/BN

where TN and BN are the Euclidean distances between the nuclear envelope (*N*), the telomere (*T*) mass centres and the intersection point of the *N–T* line with the nuclear envelope surface (*B*), respectively. Accordingly, small NRD values indicate a nuclear‐central telomere location, whereas values close to 1 correspond to the nuclear periphery.

## Conflict Of Interest

The authors have no conflicts of interest to report.

## Supporting information


**Appendix S1.** Telomere FISH on *N. benthamiana* chromosomes.

**Appendix S2.** Telomere FISH on *N. benthamiana* wild‐type interphase nucleus.

**Appendix S3.** Live interphase nucleus of *N. benthamiana* showing telomeres (Sp‐dCas9‐mRuby) and nuclear envelope (pUL50‐GFP).

**Appendix S4.** Dynamic imaging of telomeres by CRISPR–dCas9.

**Appendix S5.** 3D telomere localization.

**Appendix S6.** Telomere tracking.

**Appendix S7.** Registration of telomere movements to reference system.

**Appendix S8.** Primers used for T‐DNA construction.
