# Supplementary figures and images for: Live‐cell CRISPR imaging in plants reveals dynamic telomere movements
Source: Plant J. 2017 Jul 14;91(4):565–73. doi: 10.1111/tpj.13601 (PMC5599988; doi:10.1111/tpj.13601)

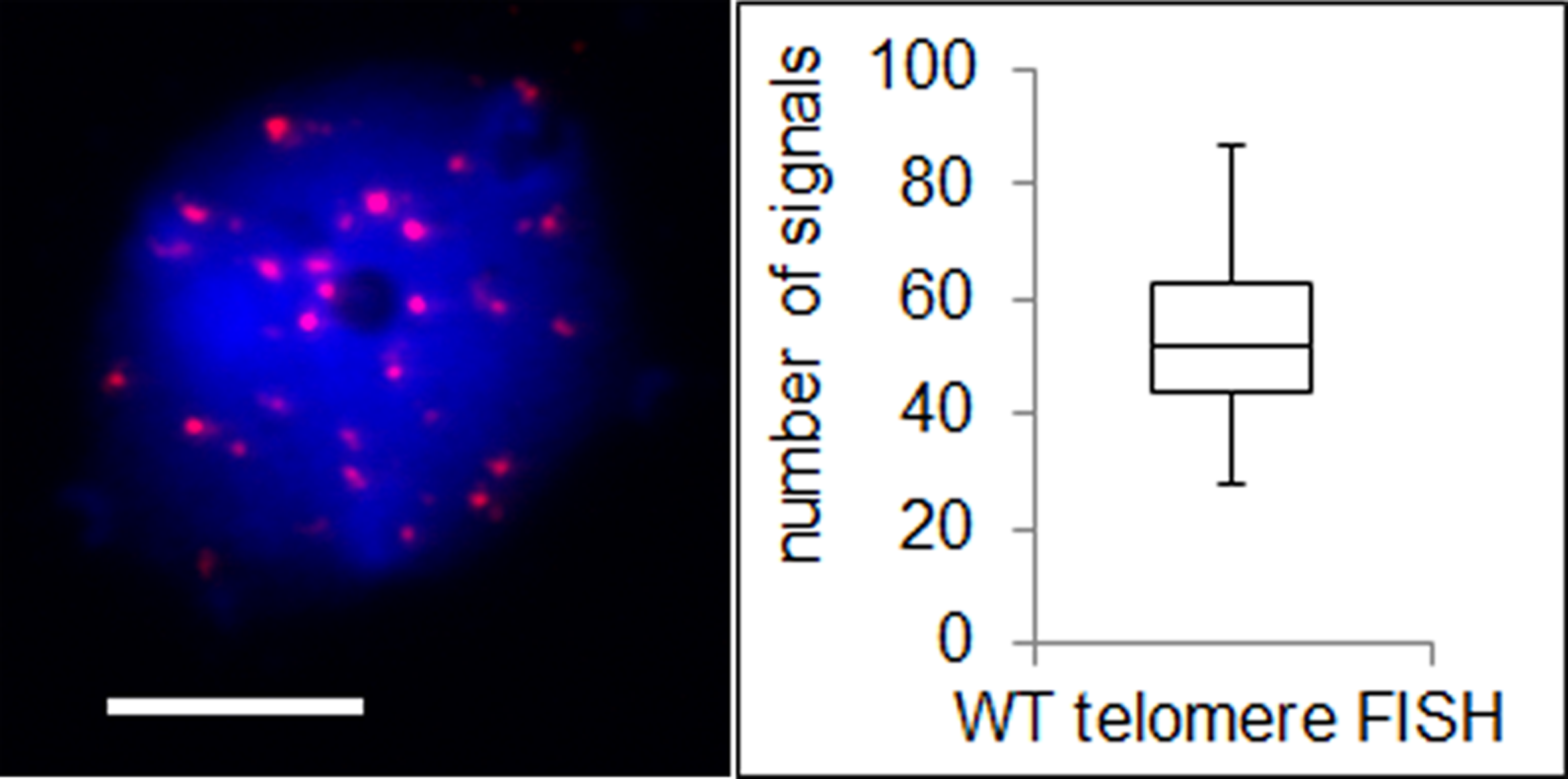

Supplement: Supplementary file 2 — Appendix S2. Telomere FISH on N. benthamiana wild‐type interphase nucleus. [file TPJ-91-565-s002.tif]
