# Supplementary material for: Live‐cell CRISPR imaging in plants reveals dynamic telomere movements
Source: Plant J. 2017 Jul 14;91(4):565–73. doi: 10.1111/tpj.13601 (PMC5599988; doi:10.1111/tpj.13601)
Supplement: Supplementary file 9 [file TPJ-91-565-s009.docx]

**Supplementary File 1 Telomere FISH on *N. benthamiana* chromosomes**

Telomere FISH probe (5’-Cy5-GGGTTTAGGGTTTAGGGTTT-3’) was applied on *N. benthamiana* chromosomes showing correct telomeric localization of our FISH probe. Scale bar equals 10 µm.

**Supplementary File 2 Telomere FISH on *N. benthamiana*wild-type interphase nucleus.** Telomere FISH probe (5’-Cy5-GGGTTTAGGGTTTAGGGTTT-3’) was applied on wild-type *N. benthamiana* interphase leaf nuclei to compare telomere localization pattern against transiently transformed leaf nuclei. Scale bar equals 10 µm. Whisker-box-plot shows the number of telomere signals of 31 nuclei.

**Appendix S3.** Live interphase nucleus of *N. benthamiana* showing telomeres (Sp-dCas9-mRuby) and nuclear envelope (pUL50-GFP)

**Supplementary File 4 Dynamic Imaging of telomeres by CRISPR-dCas9**

Telomeres were visualized by CRISPR-dCas9 (red). Nuclear envelope was visualized by pUL50-GFP (green). Total duration equals 30 minutes.

**Supplementary File 5 3D telomere localization**

3D projection of telomeres (CRISPR-imaging) and nuclear envelope (pUL50-GFP) showing telomeres being located in close proximity to the nuclear envelope.

**Supplementary File 6 Telomere tracking**

Telomeres were automatically detected based on a brightness quality threshold. Tracks are indicated by lines. Time is indicated by different colours (blue=beginning, red=end). Total duration equals 30 minutes.

**Supplementary File 7 Registration of telomere movements to reference system**

Relative nuclear movements (i.e. translations, rotations) were accounted for (see Experimental procedures). Telomere movements are shown over a period of 30 minutes without being biased by movements of the entire nucleus.

**Supplementary File 8 Primers used for T-DNA construction**

Primer names are given in column 1 and primer sequences are given in column 2.
